# Investigating the functions of subregions within anterior hippocampus

**DOI:** 10.1016/j.cortex.2015.09.002

**Published:** 2015-12

**Authors:** Peter Zeidman, Antoine Lutti, Eleanor A. Maguire

**Affiliations:** a Wellcome Trust Centre for Neuroimaging, Institute of Neurology, University College London, London, UK; b Centre Hospitalier Universitaire Vaudois, Lausanne, Switzerland

**Keywords:** Anterior hippocampus, Scene construction, Subfields, Episodic memory, Consolidation

## Abstract

Previous functional MRI (fMRI) studies have associated anterior hippocampus with imagining and recalling scenes, imagining the future, recalling autobiographical memories and visual scene perception. We have observed that this typically involves the medial rather than the lateral portion of the anterior hippocampus. Here, we investigated which specific structures of the hippocampus underpin this observation. We had participants imagine novel scenes during fMRI scanning, as well as recall previously learned scenes from two different time periods (one week and 30 min prior to scanning), with analogous single object conditions as baselines. Using an extended segmentation protocol focussing on anterior hippocampus, we first investigated which substructures of the hippocampus respond to scenes, and found both imagination and recall of scenes to be associated with activity in presubiculum/parasubiculum, a region associated with spatial representation in rodents. Next, we compared imagining novel scenes to recall from one week or 30 min before scanning. We expected a strong response to imagining novel scenes and 1-week recall, as both involve constructing scene representations from elements stored across cortex. By contrast, we expected a weaker response to 30-min recall, as representations of these scenes had already been constructed but not yet consolidated. Both imagination and 1-week recall of scenes engaged anterior hippocampal structures (anterior subiculum and uncus respectively), indicating possible roles in scene construction. By contrast, 30-min recall of scenes elicited significantly less activation of anterior hippocampus but did engage posterior CA3. Together, these results elucidate the functions of different parts of the anterior hippocampus, a key brain area about which little is definitely known.

## 1 Introduction

Little is known about the functional anatomy of the anterior, or head, of the human hippocampus. It is a complex brain region with unique cellular morphology, positioned at the apex of the amygdala, parahippocampal gyrus (including entorhinal cortex) and posterior hippocampus (Ding and Van Hoesen, 2015, Poppenk et al., 2013, Strange et al., 2014). Few neuroimaging studies have focussed on the detailed anatomy of anterior hippocampus, with most having insufficient spatial resolution. The complexity of this region is reflected in the wide variability across protocols for delineating its substructures from MRI scans (Yushkevich et al., 2015). However, understanding the contribution of structures within anterior hippocampus may offer new insights into the spectrum of deficits in patients with hippocampal lesions, as well as better explaining the role of the hippocampus in everyday cognition.

Currently, we know that anterior hippocampus is engaged during functional MRI (fMRI) scanning when participants recall their personal past experiences, or autobiographical memories (e.g., Addis et al., 2012, Addis et al., 2009, Addis et al., 2007, Bonnici, Chadwick, Lutti, et al., 2012, Hassabis, Kumaran, & Maguire, 2007). But recall of the past is not essential to activate anterior hippocampus. Imagining or simulating future events also engages this region (e.g., Addis et al., 2011, Addis et al., 2009, Addis et al., 2007), as does constructing fictitious scenes or events in the imagination without a temporal dimension (Hassabis, Kumaran, & Maguire, 2007, Zeidman et al., 2014). Indeed, a variety of fMRI studies involving scene stimuli have documented engagement of anterior hippocampus. For instance, Poppenk, McIntosh, Craik, and Moscovitch (2010) found that anterior hippocampus responded to novel relative to familiar subsequently-remembered scenes. Scene novelty was also investigated by Howard, Kumaran, Olafsdottir, and Spiers (2011), who manipulated the relative placement of objects, backgrounds and whole scenes presented visually. They found anterior hippocampus was maximally activated when changing the position of an object with respect to its background, thereby altering the spatial configuration of the scene. Zeidman et al. (2015) found that viewing scenes without an explicit task is sufficient to evoke anterior hippocampal activation. It seems, therefore, that the anterior hippocampus may be particularly responsive to scenes. Moreover, patients with bilateral damage to the hippocampi, including the anterior portion, are impaired at constructing internal representations or models of scenes (e.g., Hassabis, Kumaran, Vann, et al., 2007, Kurczek et al., 2015, Mullally et al., 2012, Race et al., 2011).

Closer inspection of the fMRI literature reveals a further intriguing observation. The increased fMRI activity for scenes and events appears to be localised to a specific region of anterior hippocampus, in the medial rather than the lateral portion of the structure, which we refer to as anterior medial hippocampus (amHipp; Zeidman et al., 2015). Anterior hippocampus consists of the intraventricular (lateral) and extraventricular (medial) parts (Duvernoy, 1988). The intraventricular portion consists of the subfields (plus the subiculum), a well-known circuit including regions dentate gyrus (DG), CA3, CA2, CA1, subiculum (Sub) and presubiculum/parasubiculum (PrS/PaS). The extraventricular part, also called the uncus, is particularly relevant here because of its medial aspect. The uncus, anterior Sub and PrS/PaS could be strong candidates for the source of activity in amHipp due to their location and connectivity with other brain regions that respond to scenes and autobiographical memory (Ding, 2013, Insausti and Munoz, 2001) – the so-called ‘core’ network, which includes parahippocampal, retrosplenial and ventromedial prefrontal cortices (Addis et al., 2009, Andrews-Hanna et al., 2010, Summerfield et al., 2010, Svoboda et al., 2006).

Here, we first aimed to identify the subfields of the hippocampus that are engaged by scenes, providing a better understanding of why many studies using scene stimuli have found activation in amHipp. We had participants imagine novel scenes while undergoing fMRI, as well as recalling scenes that were encoded thirty minutes or one week before scanning. Matched baseline tasks using single isolated objects instead of scenes served as control conditions, and we compared the response to scenes against the response to objects in each substructure of the hippocampus. We did this by capitalising on advances in high resolution structural MRI and fMRI, and extended and refined our existing hippocampal subfield segmentation protocol (Bonnici, Chadwick, Kumaran, et al., 2012) to increase the precision of our analyses around amHipp. Specifically, we separately defined the uncus from the main section of anterior hippocampus, as well as including PrS/PaS which had not previously been part of the protocol. We suspected involvement of these regions due to their medial aspect within the hippocampus.

Our second aim was to compare the hippocampal response to imagining novel scenes against the response to recalling scenes from the past. It has previously been demonstrated that imagination of novel scenes and recall of autobiographical memories both engage anterior hippocampus (Hassabis, Kumaran, & Maguire, 2007). In a recent study, Bonnici, Chadwick, and Maguire (2013) compared autobiographical memory recall from 2 weeks prior to scanning to memory recall from 10 years ago, and found both could be decoded in the subfields of the anterior hippocampus. Thus, anterior hippocampus may be recruited when constructing a scene from representations distributed across cortex – whether novel or based on specific memories which have undergone systems consolidation (Marr, 1971, Squire, 1992, Squire and Wixted, 2011). Here, we investigated recall at a more fine-grained timescale than Bonnici et al. (2013). We hypothesised that imagining novel scenes and recalling scene memories from one week earlier would engender a similar response in the hippocampus, because in both conditions, information distributed across cortex must be assembled into a coherent representation. Beyond the hippocampus, we expected both imagination and 1-week recall of scenes to recruit the same regions for translating the internal representation into a vivid sensory experience, including parahippocampal cortex (PHC) and retrosplenial cortex (RSC) (Byrne, Becker, & Burgess, 2007).

By contrast, we hypothesised a distinct profile of results for recalling very recent scene memories that had been formed just 30 min before scanning. As these had not yet undergone systems consolidation, we expected less demand on the process of scene construction, and thus reduced hippocampus activation. Note that this is the opposite viewpoint to standard systems consolidation theory, which states that memories become more independent of the hippocampus over time (Squire, 1992). In common with the constructing novel scenes and recalling scenes from a week earlier, the 30-min recall task required subjects to produce simulated sensory experience, and we expected this to be reflected by engagement of PHC and RSC.

## 2 Materials and methods

### 2.1 Participants

There were eighteen healthy, right-handed participants (6 males, mean age 23.17 years, SD 3.31, range 19–30). All had normal or corrected-to-normal vision, and gave informed written consent to participation in accordance with the University College London Research Ethics Committee.

### 2.2 Task and procedure

**Fig. 1:**
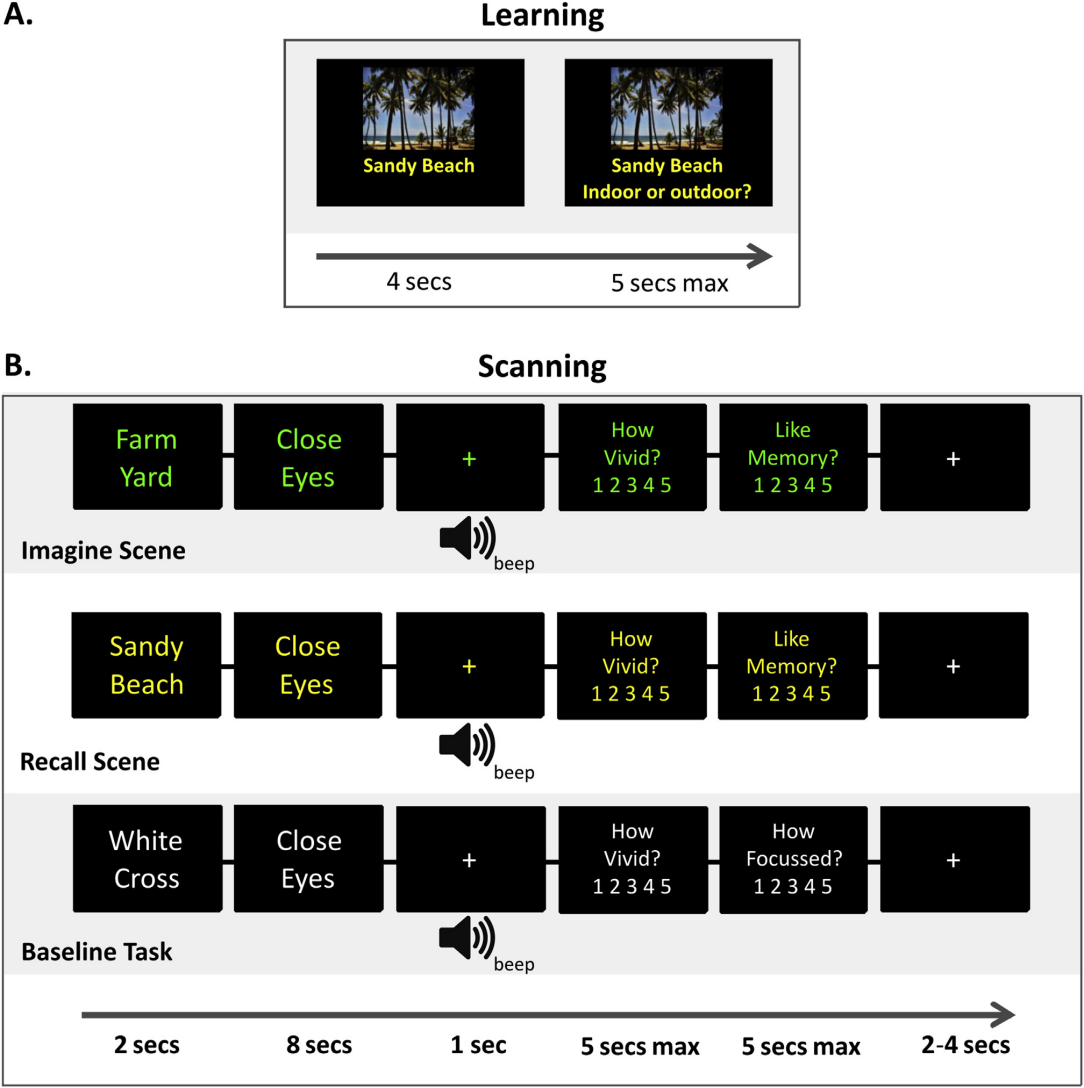
**Experimental paradigm**. **A**. The pre-scan learning phase. Participants viewed each stimulus for 4 sec, accompanied by a 2-word description. They were then presented with a question while the stimulus remained visible, with a maximum response time of 5 sec. Scene and object stimuli were intermixed. **B**. **Top row**: An Imagine Scene trial during scanning. A 2-word cue is given describing the scene to be imagined. Participants then had 8 sec with their eyes closed to construct the scene in their mind's eye, before hearing a beep. They then gave two ratings – ‘vividness’ and ‘memoryness’ (see text). The ITI was jittered between 2 and 4 sec. **Middle row**: An example Recall Scene trial, where the two-word cue matches the caption from a scene viewed one week or 30 min before scanning. **Bottom row**: Baseline fixation task in which subjects had to imagine a small white fixation cross. Object conditions matched the imagine and recall examples shown here, except the cues described single objects rather than whole scenes. Beach photo credit: FlaviaC, Wikimedia Commons.

Participants made two visits to the laboratory, 7 days apart. On their first visit, they performed a 10-min fMRI task completely unconnected to this experiment, before undergoing high resolution structural MRI scanning for an hour. (No functional scanning for this experiment was conducted on their first visit.) After structural scanning they had a break, and then sat at a desktop computer to undertake learning for the experiment presented here. Stimuli were photographs of scenes and photographs of single objects presented on a plain white background. Objects were used because they were matched for the task requirements but, unlike scenes, imagining objects has not been found to be hippocampal dependent (e.g., Hassabis, Kumaran, & Maguire, 2007, Hassabis, Kumaran, Vann, et al., 2007, Hassabis and Maguire, 2007). These pictures were shown one at a time, with a two word caption underneath which described the stimulus (Fig. 1A). Participants were instructed to remember both the picture and the caption for a memory test the following week. Each stimulus was shown for 4 sec. The stimuli were shown five times each with the order of stimuli uniquely randomized for each participant. We based the number of repetitions on pilot data, which suggested that five repetitions was sufficient for reliable subsequent recall one week later. To ensure attention during encoding, each time a stimulus was shown 1 of 5 questions was asked: “Caption match the picture?”, “Indoor or outdoor?”, “Mainly man-made or natural”, “Find this scene/object in this country?” and “Does the picture contain anything red?”. All questions were asked for each stimulus over the course of the five learning trials, and were displayed underneath the stimulus after it had appeared alone on screen for 4 sec. The picture remained on screen while the question was presented and participants then had a maximum of 5 sec to respond using a button press. There were 20 scene stimuli and 20 object stimuli, plus 2 stimuli of each category which had incorrect captions – these were used as lures for the “Caption match the picture?” question. The lures and associated photographs were not used in subsequent scanning.

On their second visit one week later, 30 min prior to fMRI scanning participants learnt a new set of 20 scene and 20 object photographs (plus 2 lures), using the same procedure as their first visit. They were then trained and given practice on the scanning task (Fig. 1B), which proceeded as follows. There were 3 scene conditions (imagine scenes, recall 1-week-old scenes, recall 30-min-old scenes), 3 matched object conditions using single isolated objects instead of scenes, and a fixation baseline condition. The order of trials was pseudo-randomised across participants. In the imagine scene/object conditions, participants were given a two-word cue (in green lettering) describing the scene or object they had to construct in their imagination. They then saw the cue “close eyes”, and had 8 sec in which to construct the scene/object. They had been trained to keep their imagined viewpoint fixed, and to attempt to come up with something new rather than previous memories. An auditory beep alerted them to open their eyes, and two self-paced ratings (5 sec max) were taken. “How vivid?” [1–5, max 5] asked how vivid was the scene/object they had constructed. “Like memory?” [1–5, max 5] asked how similar it was to a specific memory.

In the recall 1-week and recall 30-min conditions, the two word cue displayed at the beginning of the trial (with yellow lettering) had been previously seen as a caption under a photograph during learning. Participants spent the 8 sec after the “close eyes” cue bringing back into their mind's eye the relevant scene or object. Their training emphasised they should not change the stimulus in any way or use their imagination, but just to spend the time focussing on bringing back what they remembered. As before, a rating for vividness was then taken, and participants responded to the “Like memory?” question with how accurately they felt their memory reproduced the original stimulus. If participants could not remember the stimulus at all, they had been trained not to imagine something new, but rather select 1 out of 5 for vividness and 1 out of 5 for “Like memory?”. These trials were then excluded from further analysis.

In the fixation baseline condition, participants were given the cue “white cross”, and had been trained to imagine a small white fixation cross and focus on it for the 8 sec. They then gave a vividness rating, and the “Like memory?” question was replaced with “How focussed?”. Participants responded with the extent to which they had maintained focus during the trial. This baseline was designed to go some way to controlling for the task demands, such as reading and understanding the cue, and maintaining attention.

Immediately on being removed from the scanner, participants were given surprise memory tests to assess whether they could remember the photograph stimuli and the two-word scene construction cues. In the test for the photographic stimuli, they were shown all 80 scene and object stimuli learnt prior to scanning, plus 40 lures they had not seen before (20 new scenes and 20 new objects), and were asked to indicate with a button press “Remember” or “Do not remember” followed by a confidence rating (1–5, max 5). Timing was self-paced with a maximum of 5 sec per stimulus. An identical memory test was then administered for the two-word cues they had seen for scene/object construction during scanning. Participants were shown all 40 two-word cues that had been used during the imagine conditions during scanning, plus 20 lures (10 scenes and 10 objects) which they had not seen before. Finally, participants answered a series of questions about the cognitive strategies they used during scanning – these are detailed in the behavioural results.

### 2.3 Image acquisition

Structural and functional data were acquired using a 3T Siemens Trio MRI scanner (Erlangen, Germany). Both types of scan were performed within a partial volume centred on the medial temporal lobe, which enabled the images to be of sufficiently high resolution to delineate the hippocampal subfields (functional scans had a 1.5 mm^3^ isotropic voxel size and structural scans had a 0.5 mm^3^ isotropic voxel size).

Structural images were collected using a single-slab 3D T2-weighted turbo spin echo sequence with variable flip angles (SPACE) (Mugler et al., 2000) in combination with parallel imaging, to simultaneously achieve a high image resolution of ∼500 μm, high sampling efficiency and short scan time while maintaining a sufficient signal-to-noise ratio (SNR). After excitation of a single axial slab the image was read out with the following parameters: resolution = .52 × 0.52 × .5 mm^3^, matrix = 384 × 328, partitions = 104, partition thickness = .5 mm, partition oversampling = 15.4%, field of view = 200 × 171 mm^2^, TE = 353 msec, TR = 3200 msec, GRAPPA ×2 in phase-encoding (PE) direction, bandwidth = 434 Hz/pixel, echo spacing = 4.98 msec, turbo factor in PE direction = 177, echo train duration = 881, averages = 1.9. For reduction of signal bias due to, for example, spatial variation in coil sensitivity profiles, the images were normalized using a prescan, and a weak intensity filter was applied as implemented by the scanner's manufacturer. To improve the SNR of the anatomical image, four scans (taking ∼12 min each) were acquired for each participant, coregistered and averaged. Additionally, a whole brain 3D FLASH structural scan was acquired with a resolution of 1 × 1 × 1 mm.

Functional data were acquired using a 3D echo planar imaging (EPI) sequence which has been demonstrated to yield improved BOLD sensitivity compared to 2D EPI acquisitions (Lutti, Thomas, Hutton, & Weiskopf, 2013). Image resolution was 1.5 mm^3^ isotropic and the field-of-view was 192 mm in-plane. Forty slices were acquired with 20% oversampling to avoid wrap-around artefacts due to imperfect slab excitation profile. The echo time (TE) was 37.30 msec and the volume repetition time (TR) was 3.65 sec. Parallel imaging with GRAPPA image reconstruction (Griswold et al., 2002) acceleration factor 2 along the PE direction was used to minimize image distortions and yield optimal BOLD sensitivity. The dummy volumes necessary to reach steady state and the GRAPPA reconstruction kernel were acquired prior to the acquisition of the image data as described in Lutti et al. (2013). Correction of the distortions in the EPI images was implemented using B0-field maps obtained from double-echo FLASH acquisitions (matrix size 64 × 64; 64 slices; spatial resolution 3 mm^3^; short TE = 10 msec; long TE = 12.46 msec; TR = 1020 msec) and processed using the FieldMap toolbox available in SPM (Hutton et al., 2002).

### 2.4 Behavioural data analysis

Data from the post-scan memory test and interview were analysed using repeated measures ANOVAs and paired sample *t*-tests (SPSS 17.0, Chicago: SPSS Inc.) with a significance threshold of *p* < .05.

### 2.5 Scanning data analysis

FMRI data were analysed using SPM12 (www.fil.ion.ucl.ac.uk/spm). All images were first bias corrected to compensate for image inhomogeneity associated with the 32 channel head coil (Van Leemput, Maes, Vandermeulen, & Suetens, 1999). Fieldmaps had been collected and were used to generate voxel displacement maps. EPIs for each session were then realigned to the first image and unwarped using the voxel displacement maps calculated above. The four high-resolution structural images were averaged to reduce noise, and co-registered to the whole brain structural scan. EPIs were also co-registered to the whole brain structural scan.

**Fig. 2:**
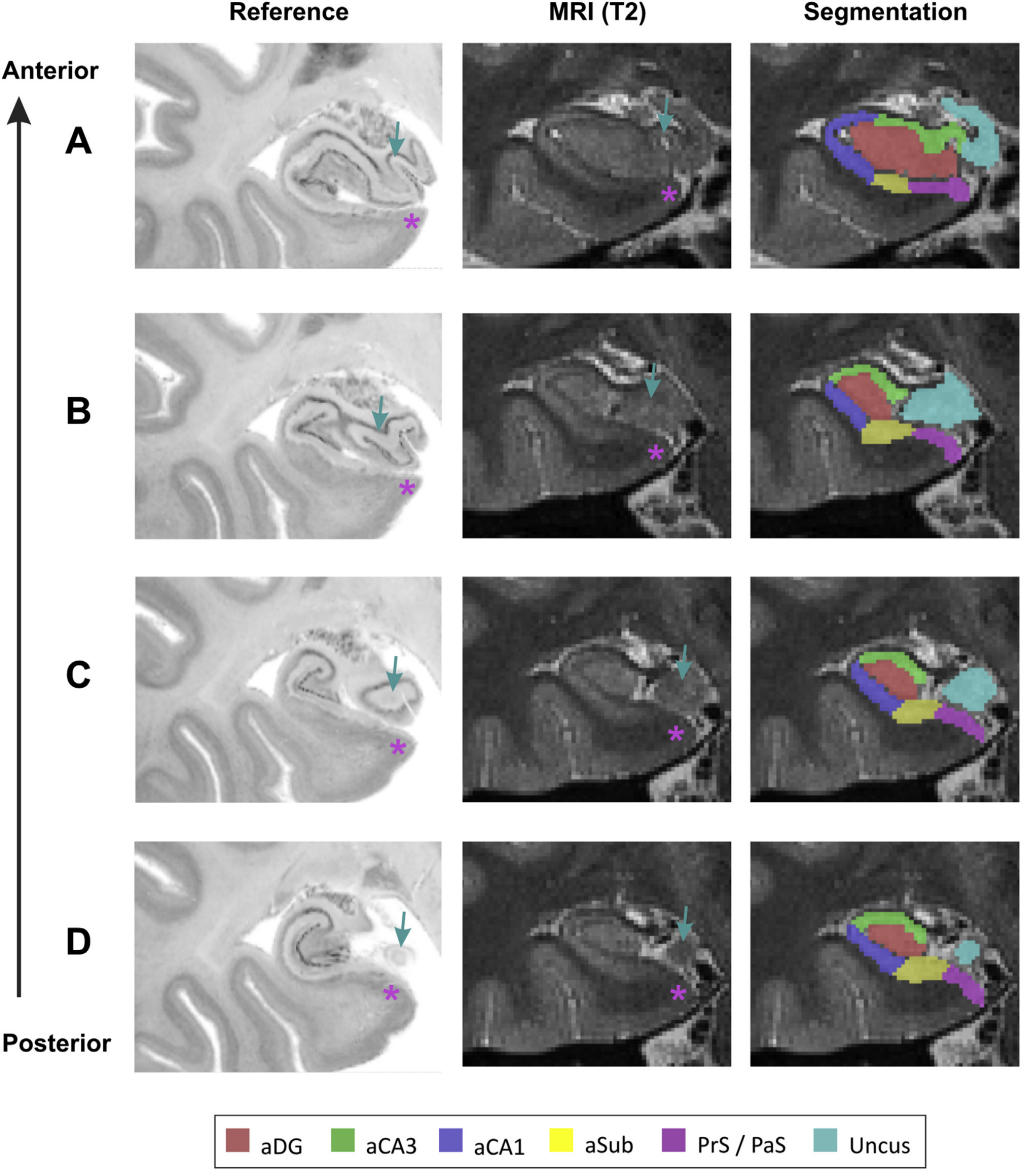
**Segmentation protocol for anterior hippocampus**. Coronal slices are ordered from anterior (A) to posterior (D). **A**. Slice including the anterior portion of the uncus of the hippocampus (the band of Giacomini). **B-D**. Slices showing separation of the uncus from the intraventricular (lateral) hippocampus. The disappearance of the uncus after slice D defines the rear-most slice of anterior hippocampus. **Left column**: Nissl stained post-mortem slices from the BigBrain project (Amunts et al., 2013). **Middle column**: slices from a single participant's T2 structural MRI in this study. **Right column**: example manual segmentation of these slices. aDG (red) = anterior dentate gyrus/CA4, aSub (yellow) = anterior subiculum, PrS/PaS (purple) = presubiculum/parasubiculum. Arrows indicate the uncus and asterisks indicate PrS/PaS.

We manually segmented the subfields of the hippocampi using the high-resolution structural image (resolution ∼0.5 mm^3^) for each subject, following the protocol of Bonnici, Chadwick, Kumaran, et al. (2012) with reference to Duvernoy (1988). We modified the protocol for anterior hippocampus as follows (Fig. 2). Segmentation of the uncus began in the first coronal slices where DG was observed and covered the band of Giacomini (Fig. 2A). The anterior-most section of the uncus, which is associated with the amygdala, was not included. At this level DG was clearly visible, and dorsal to this a hypo-intensity in the T2 signal and narrowing of the gyrus was identified as the transition from CA3 to CA1. More posteriorly the uncus was seen to separate from intraventricular hippocampus, sitting alongside it (Fig. 2B and C). The most posterior slice to include the uncus before it disappeared from view (Fig. 2D) marked the posterior boundary of anterior hippocampus. In these slices, the CA3/CA1 border was placed at the ‘shoulder’ of the gyrus where it began to narrow towards CA1. The definition of the subiculum in anterior (a) hippocampus was adjusted as follows. The aCA1/aSub border was positioned ventral to aDG guided by the study of Ding (2013). A marked hypo-intensity in the T2 signal marked the border between aSub and PrS/PaS. Following the analysis by Fischl et al. (2009), PrS/PaS went no further ventrally than the ‘shoulder’ of the gyrus in order avoid inclusion of entorhinal cortex. Images were segmented using ITK-SNAP (Yushkevich et al., 2006) and a graphics tablet. Segmented masks were then resliced to 1.5 mm isotropic voxels to match the functional images using SPM (with 4th degree B-Spline interpolation). Masks for DG, CA3, CA1 and Sub were then divided into anterior (a) and posterior (p) portions as described above.

**Fig. S1:**
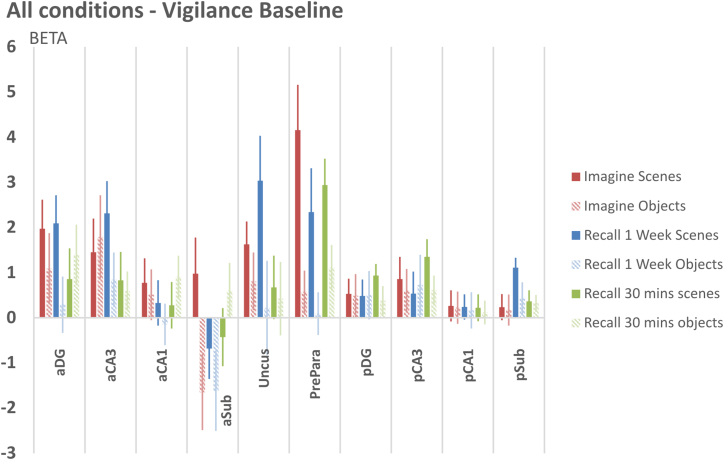
**All responses versus the fixation baseline task**. The plot shows parameter estimates for all experimental conditions (scenes and objects) versus the fixation baseline task in each region of interest. aDG = anterior dentate gyrus, aCA3 = anterior CA3, aCA1 = anterior CA1, aSub = anterior subiculum, PrS/PaS = presubiculum/parasubiculum, pDG = posterior dentate gyrus, pCA3 = posterior CA3, pCA1 = posterior CA1, pSub = posterior subiculum. ±1 SEM.

For the regions of interest (ROI) analyses, functional data were analysed at the single subject level without warping or smoothing. We built a general linear model (GLM) for each subject with 7 task regressors (imagine scenes, recall 1-week scenes, recall 30-min scenes, imagine objects, recall 1-week objects, recall 30-min objects, fixation task). Each condition was modelled from the onset of the cue to just before the beep sounded indicating the eyes should be opened (10 sec). Additional regressors modelled the beep (as a zero length event) and the ratings period. The ITI was not modelled and so acted as the implicit baseline. Two further regressors modelled the BOLD signal obtained from ROIs in the white matter and CSF. Following estimation of the GLMs, each subject's mask image was inspected for dropout in the hippocampus. In some subjects, voxels were excluded by SPM, which was traced back to single volumes with large spikes. These were repaired by averaging the volumes on either side and modelling any repaired volumes using an extra regressor in that subject's design matrix. To perform the ROI analyses we extracted the mean value from the contrast image for each condition (relative to the implicit baseline). We used *t*-tests to evaluate significance at the group level. All results presented here are for scene conditions with object baselines subtracted. Parameter estimates for each condition individually versus the fixation baseline task are provided in the supplementary material (Fig. S1).

For group level analyses we created a group DARTEL template (Ashburner, 2007) using the whole brain structural scans from each participant's first visit. Functional images were warped via the group space to MNI space, then spatially smoothed using a Gaussian smoothing kernel of 4 × 4 × 4 mm full-width at half maximum, which we found to be the minimum to satisfy the smoothness assumptions of random field theory-based multiple comparisons correction in SPM. GLMs were created as above (with one modification – the fixation baseline condition was left unmodelled). After estimating the GLMs we summarised results at the group level using one sample *t*-tests in SPM. Where conjunctions are reported, these are tests against the conjunction null, that is to say a logical AND across conditions. Results are reported using topological FDR correction at *p* < .05 with cluster forming threshold of *p* < .01 unless stated otherwise.

## 3 Results

### 3.1 Behavioural results

#### 3.1.1 Performance during scanning

After recalling or imagining each scene or object in the scanner, participants gave two ratings. The first was vividness [1–5, max 5]. If participants felt they had entirely failed to imagine or recall a scene, they had been trained to indicate this by responding with 1 out of 5. Counting the number of failed and non-failed trials in each condition gave a first measure of success. The mean success rate (trials with vividness of more than 1 out of 5) was over 85% in all conditions (imagine scenes: mean 98.89%, SD 3.66%; recall 1-week scenes: mean 87.22%, SD 8.78%; recall 30-min scenes: mean 97.78%, SD 6.00%; imagine objects: mean 95.83%, SD 7.33%; recall 1-week objects: mean 86.67%, SD 8.74%; recall 30-min objects: mean 97.22%, SD 3.92%). A repeated measures ANOVA with factors of stimulus type (scene or object) and condition (imagine, recall 1-week stimuli, recall 30-min stimuli) did not show a significant difference in success rate between scene and object trials [*F*(1,17) = 2.46, *p* = .14], but there was a difference between conditions [*F*(2,34) = 27.86, *p* = 6.86e-8] with no significant interaction between factors [*F*(2,34) = .70, *p* = .50]. Post-hoc *t*-tests showed that the success rate of the 1-week recall scenes condition was significantly less than the recall 30-min scenes condition [*t*(17) = 4.19, *p* = .001] and less than the imagine scenes condition [*t*(17) = 5.18, *p* = 7.5e-5]. The same pattern of results was observed in the object conditions: success for 1-week objects was significantly less than 30-min objects [*t*(17) = 4.86, *p* = 1.48e-4] and imagining objects [*t*(17) = 3.93, *p* = .001]. Failed trials (vividness rating of 1) were removed from further behavioural analyses and regressed out in the fMRI analyses.

We next examined the vividness ratings from successful trials (rated at least 2 out of 5). A repeated measures ANOVA with factors of stimulus types (scene or object) and condition (imagine, recall 1-week stimuli, recall 30-min stimuli) showed there was no significant difference in vividness between scenes and objects [*F*(1,17) = 1.69, *p* = .21], but there was a significant difference across experimental conditions [*F*(2,34) = 26.90, *p* = 9.89e-8]. The interaction between stimulus type and condition was not significant [*F*(2,34) = 1.76, *p* = .19]. The 30-min recall condition was rated as the most vivid of the three conditions [scenes: mean 4.41 (SD .54), objects: mean 4.49 (SD .40)]. Slightly less vivid were the recall 1-week conditions [scenes: mean 3.84 (SD .51), objects: mean 3.80 (SD .69)] and imagine conditions [scenes: mean 3.90 (SD .59), objects: mean 4.12 (SD .53)]. Post-hoc *t*-tests showed 30-min scenes were significantly more vivid than 1-week scenes [*t*(17) = 6.73, *p* = 4e-6] and imagined scenes [*t*(17) = 5.56, *p* = 3.5e-5]. Similarly, vividness was significantly higher for 30-min objects than 1-week objects [*t*(17) = 5.21, *p* = 7.1e-5] and imagined objects [*t*(17) = 4.07, *p* = .001]. There was no significant difference in vividness between imagined scenes and 1-week recalled scenes [*t*(17) = .60, *p* = .56], whereas imagined objects were significantly more vivid than objects recalled from one week earlier [*t*(17) = 2.25, *p* = .04].

The second rating given after every imagine or recall trial was ‘memoryness’ (range 1–5, max 5). It was intended that imagined scenes/objects should be the least like memories (with low ratings), whereas recalled scenes/objects should be complete and accurate memories (with high ratings). A repeated measures ANOVA with factors of stimulus type (scene or object) and condition (imagine, recall 1-week stimuli, recall 30-min stimuli) showed no difference in memory ratings between scenes and objects [*F*(1,17) = .21, *p* = .65], but there was a significant difference across conditions [*F*(2,34) = 62.35, *p* = 4.22e-12]. The interaction was not significant [*F*(2,34) = .50, *p* = .61]. Post-hoc *t*-tests showed that, as intended, imagined scenes were rated as being the least similar to memories (mean 2.43, SD .62), recalled 1-week scenes were more like memories (mean 3.64, SD .60) and recalled 30-min scenes had the highest rating (mean 4.49, SD .35). Post-hoc *t*-tests showed each difference to be significant [recall 30-min scenes > imagine scenes: *t*(17) = 11.95, *p* = 1.08e-9]; recall 30-min scenes > recall 1-week scenes: *t*(17) = 6.33, *p* = 8e-6; recall 1-week scenes > imagine scenes: *t*(17) = 5.39, *p* = 4.9e-5]. The same pattern was observed for the object conditions, with imagined objects rated least similar to memories (mean 2.39, SD .67), recalled 1-week objects more similar to memories (mean 3.55, SD .69) and the highest rating was for recalled 30-min objects (mean 4.53, SD .36). These differences were statistically significant [recall 30-min objects > imagine objects: *t*(17) = 11.00, *p* = 3.77e-9; recall 30-min objects > recall 1-week objects: *t*(17) = 6.54, *p* = 5e-6; recall 1-week objects > imagine objects: *t*(17) = 4.12, *p* = .001].

To summarise the in-scanner ratings, subjects indicated they were successful in the majority of trials. Vividness was significantly higher for scenes viewed 30 min prior to scanning than those viewed a week earlier or newly imagined and this was also reflected in the corresponding object conditions. Memoryness ratings showed that imagined scenes were significantly less similar to memories than recalled scenes. There was also a difference between recall conditions, but this was matched between scenes and objects. The subtraction of the object conditions from the scene conditions in the fMRI analyses therefore goes some way toward controlling for any qualitative differences such as vividness and similarity to memories between conditions.

#### 3.1.2 Post-scan memory test

Following scanning, participants were given two surprise memory tests – one to evaluate whether they remembered the scenes or objects they had been asked to recall during scanning, and the other to evaluate if they remembered the two-word cues from the imagine trials. Failure to remember any stimuli/cues indicated that the stimuli had not been encoded during scanning and/or training. These trials were then excluded from the fMRI analysis.

Beginning with the test for memory of imagine cues, we collated the scores and calculated True Positive (TP), True Negative (TN), False Positive (FP) and False Negative (FN) rates. Performance was near ceiling (Table 1). Repeated measures ANOVAs each with a single factor of stimulus (scenes or objects) showed no significant effect of stimulus on TP [*F*(1,17) = .80, *p* = .38], TN [*F*(1,17) = .02, *p* = .88], FP [*F*(1,17) = .10, *p* = .92] or FN [*F*(1,17) = .42, *p* = .53]. To analyse the results for the scene recall conditions we used random effects ANOVAs, each with factors of stimulus (scenes or objects) and condition (1-week recall or 30-min recall). The TP rate was again near ceiling, although there was a significant effect of stimulus [*F*(1,17) = 47.10, *p* = 3e-6] and condition [*F*(1,17) = 10.97, *p* = .004], as well as a significant interaction [*F*(1,17) = 45.88, *p* = 3e-7]. This was driven by a lower TP rate in the 1-week objects condition (mean .91, SD .07). An ANOVA on the TN rate showed only a main effect of stimulus [*F*(1,17) = 7.26, *p* = .02], with a higher rate for scenes than objects. The FP rate also showed a main effect of stimulus [*F*(1,17) = 7.04, *p* = .02], driven by more FPs for objects than scenes. Finally, the FN rate showed a significant effect of stimulus [*F*(1,17) = 29.56, *p* = 4.4e-5], condition [*F*(1,17) = 14.65, *p* = .001] and an interaction [*F*(1,17) = 37.72, *p* = 1.1e-5]. This was driven by a greater FN rate for 1-week object recall.

In summary, post-scan memory scores were near ceiling and demonstrated attention to the task. In general, performance was worst for objects recalled from one week earlier. Any scenes/objects not subsequently remembered were removed from the fMRI analyses.

#### 3.1.3 Post-scan questionnaire

Following the memory tests, participants completed a questionnaire on their experience in the scanner. They rated the difficulty of each scanning condition (difficulty rating 1–5, max 5) and the results showed they felt able to perform the tasks with ease (imagine scenes: mean 2.72, SD .96; imagine objects: mean 2.72, SD 1.18; recall 1-week scenes: mean 2.61, SD 1.33; recall 1-week objects: mean 2.56, SD 1.15; recall 30-min scenes: mean 1.28, SD .57; recall 30-min objects: mean 1.2, SD .73; fixation baseline: mean 2.56, SD 1.38). A repeated measures ANOVA with factors of condition (imagine, recall 1-week, recall 30-min) and stimulus type (scenes or objects) demonstrated a significant effect of condition [*F*(2,34) = 23.49, *p* = 3.91e-7] but no effect of stimulus type [*F*(1,17) = .02, *p* = .88] nor an interaction [*F*(2,34) = .03, *p* = .93]. While there was no significant difference in difficulty between the imagination and 1-week recall of scenes [*t*(17) = .35, *p* = .73] or objects [*t*(17) = .53, *p* = .60], the 30-min recall conditions were significantly less difficult than the other conditions [recall 1-week scenes > recall 30-min scenes: *t*(17) = 4.97, *p* = 1.16e-4; recall 1-week objects > recall 30-min objects: *t*(17) = 6.23, *p* = 9.0e-6; imagine scenes > recall 30-min scenes: *t*(19) = 5.88, *p* = 1.8e-5; imagine objects > recall 30-min objects: *t*(19) = 5.53, *p* = 3.7e-5].

Participants were asked for a single rating of whether they rehearsed the stimuli in their mind during the week before scanning (rehearsal rating 1–5, 1 = never … 5 = regularly/systematically). The mean across participants was low at 1.9 (SD .83). Participants followed the instruction to keep the imagined viewpoint in a fixed position for the majority of imagined scenes (1–5, max 5, mean 4.11, SD .67). For the imagine object condition, participants were asked to rate the extent to which they successfully imagined each object in isolation against a plain background (rating 1–5 max 5). Success rates were high with a mean rating of 4.0 (SD .84).

To summarise the post-scan ratings, participants indicated that they did not find the task too difficult. While the 30-min scene recall condition was rated as being less difficult than the other scene conditions, this was matched by the same pattern of responses for the object conditions. The ratings also gave us confidence that the stimuli recalled from a week earlier were not over-rehearsed and that our instructions for the scanning task were followed.

### 3.2 fMRI results

#### 3.2.1 ROI analysis

We used a GLM to calculate parameter estimates (betas) for each condition in each voxel in the hippocampus. We computed contrasts to subtract object baselines from the scene conditions, then averaged the contrast values within each subfield. To interrogate these data we entered the contrast values into a random effects ANOVA with factors of Task (imagine, 1-week recall, 30-min recall), Hemisphere (left or right) and Region (aDG, aCA3, aCA1, aSub, uncus, PrS/PaS, pDG, pCA3, pCA1, pSub). We found a significant main effect of Region [*F*(9,153) = 3.41, *p* = .03] and an interaction between Task and Region [*F*(18,306) = 2.85, *p* = .03]. Neither the main effect of Task [*F*(2,34) = 2.30, *p* = .12] nor Hemisphere [*F*(1,17) = .35, *p* = .56] was significant. In the remainder of this section we unpack these results to address our hypotheses. Full results of all planned post-hoc tests are provided in supplementary data Tables S1 and S2.

**Fig. 3:**
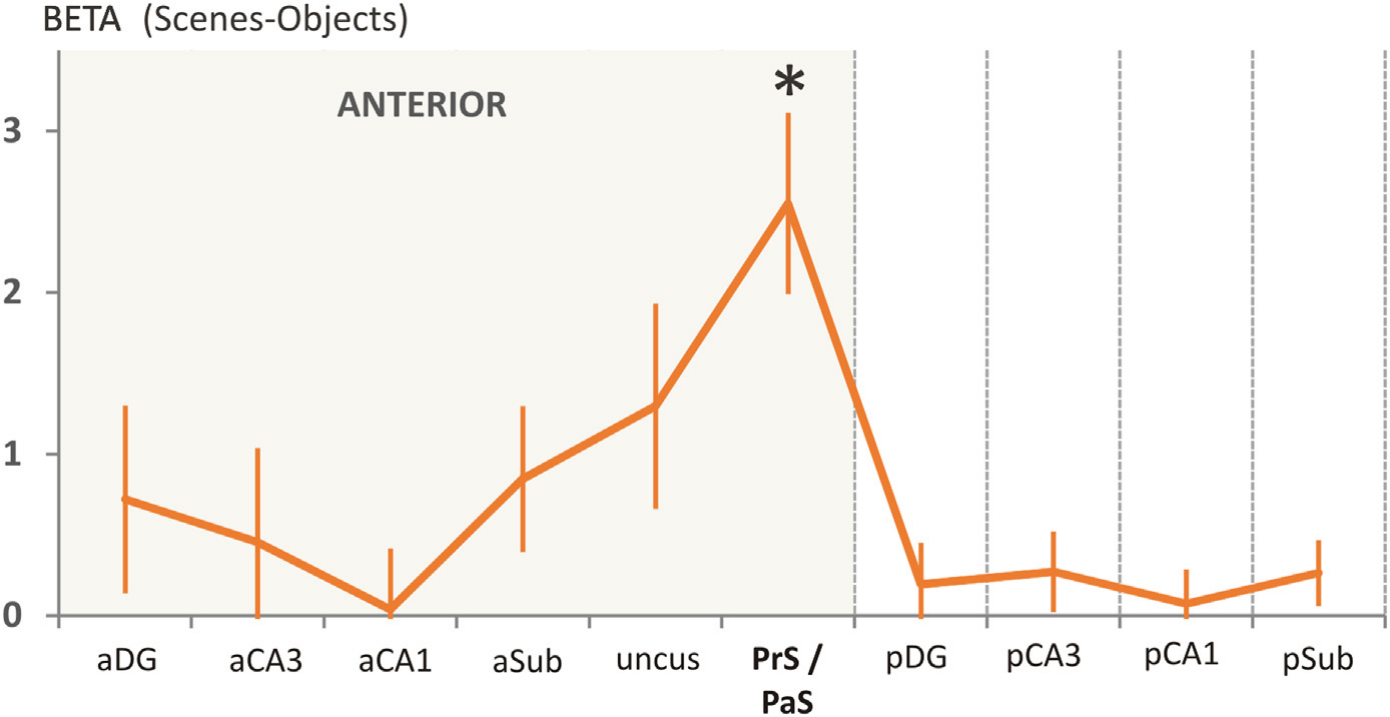
**The response of each subregion of the hippocampus to scenes**. Data are relative to object baselines and collapsed over experimental condition and hemisphere, to show the main effect of region (see text). The *y*-axis represents the difference in parameter estimates for scenes and objects, where positive values represent a stronger response to scenes than object baselines. aDG = anterior dentate gyrus, aCA3 = anterior CA3, aCA1 = anterior CA1, aSub = anterior subiculum, PrS/PaS = presubiculum/parasubiculum, pDG = posterior dentate gyrus, pCA3 = posterior CA3, pCA1 = posterior CA1, pSub = posterior subiculum. +/− 1SEM; *p < .05, one-sample *t*-test.

Our first experimental question was which hippocampal subfields are engaged by scenes, collapsed across the imagine and recall tasks. To address this we examined the main effect of Region identified in the ANOVA, collapsing over Task and Hemisphere. As expected, the effect was driven by regions in anterior hippocampus (Fig. 3), particularly aDG, aSub, uncus and PrS/PaS. The largest response was in PrS/PaS, and post-hoc *t*-tests found only PrS/PaS to have significantly stronger activation for scenes than objects [*t*(17) = 4.56, *p* = 3e-4].

**Fig. 4:**
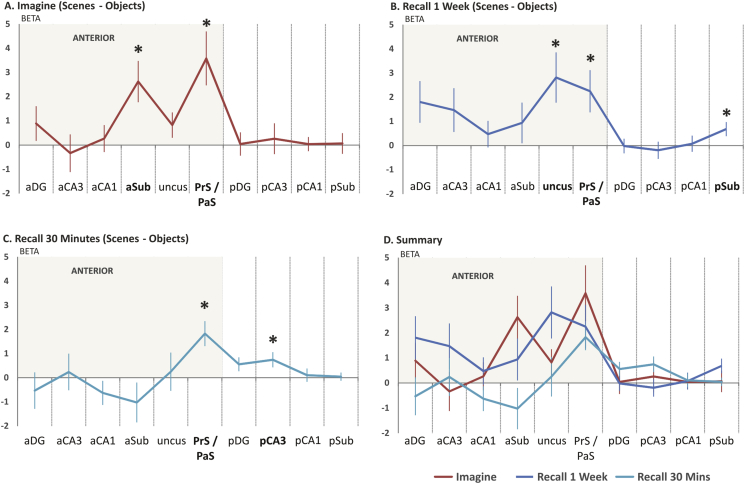
**The interaction between region and task**. Data are relative to object baselines and collapsed over hemisphere. Each *y*-axis represents the difference in parameter estimates between scenes and object baselines, where positive values represent a stronger response to scenes than objects. Graphs show **A**. imagining novel scenes **B**. recalling scenes from one week earlier **C**. recalling scenes from 30 min earlier **D**. Summary with each plot overlaid. Abbreviations as for Fig. 3. +/− 1SEM; *p < .05, one-sample *t*-test.

Our second question concerned how Task (the individual imagine and recall conditions) modulated the response to scenes in the hippocampal subfields. We investigated this by unpacking the significant 2-way interaction between Task and Region identified in the ANOVA. We hypothesised that imagining scenes and 1-week recall would give similar profiles of results, and indeed both tasks engaged subfields of anterior hippocampus (Fig. 4A and B). There were also differences between imagination and 1-week recall. In the imagine condition, PrS/PaS was accompanied by aSub (Fig. 4A), whereas in the 1-week recall condition PrS/PaS was accompanied by the uncus and pSub (Fig. 4B). Post-hoc paired samples *t*-tests confirmed the differences in aSub and uncus [aSub imagine > recall 1 week: *t*(17) = 2.28, *p* = .04; uncus recall 1 week > imagine: *t*(17) = 2.13, *p* = .05] with no significant differences between imagine and 1-week recall in the other subfields.

We hypothesised less activity in anterior hippocampus during 30-min scene recall compared to the other scene conditions as these had not yet undergone systems consolidation and so we expected less demand on the process of scene construction. The response across anterior hippocampus was clearly less than the other conditions (Fig. 4C), and post-hoc *t*-tests confirmed significantly reduced activation of aSub for 30-min recall than imagining scenes [*t*(17) = 2.65, *p* = .02] and reduced activation of the uncus for 30-min recall compared to recall from a week earlier [*t*(17) = 3.25, *p* = .005]. No anterior subfield had a significantly greater response to 30-min recall than the other conditions. Nonetheless, the hippocampus was engaged by the 30-min recall task, with significant activation of PrS/PaS [*t*(17) = 3.57, *p* = .002] and pCA3 [*t*(17) = 2.41, *p* = .03].

In summary, we used an ROI analysis to identify the subfields which underpin activation of amHipp when scenes are imagined or recalled. Three scene tasks (imagining novel scenes, 1-week scene recall and 30-min scene recall) all engaged PrS/PaS, relative to matched single-object baselines. This was complemented by aSub for imagining novel scenes and the uncus for 1-week scene recall. As expected, the 30-min scene recall condition elicited less activation of the anterior hippocampus, but was still associated with significant engagement of PrS/PaS and pCA3. Next, to understand our findings from the hippocampus in the context of the wider brain, we performed SPM analyses on the whole scanning volume.

#### 3.2.2 Whole volume analysis

##### 3.2.2.1 Main effect of scenes

**Fig. 5:**
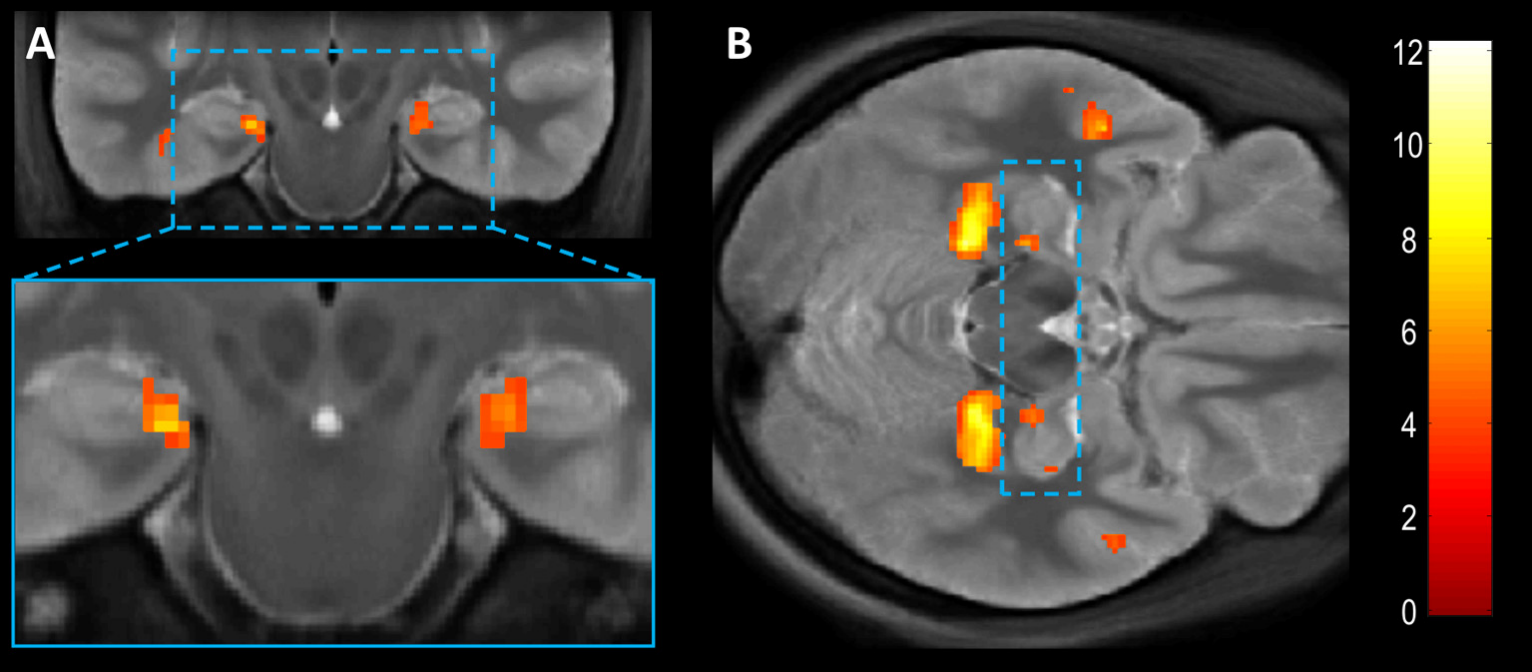
**The response to scenes in anterior medial hippocampus and the wider volume**. SPM whole-volume analysis testing for a stronger response to scenes than single isolated objects (collapsed over task). **A**. Coronal slice showing activation of anterior medial hippocampus (MNI *y* = −20). Inset, the medial temporal lobe enlarged for clarity. **B**. Axial slice showing hippocampi (blue box), PHC, STS and vmPFC. Images thresholded at *p* < .001 uncorrected for display purposes, overlaid on the group average T2 structural volume.

We began our analyses of the fMRI (partial) volume by testing for a main effect of Scenes [(scenes − objects)], in order to identify regions beyond the hippocampus which co-activated with amHipp. Reproducing the commonly observed scene network, there was greater activation for scenes than objects in amHipp, bilateral PHC, RSC, ventromedial prefrontal cortex (vmPFC) and superior temporal sulcus (STS) (Table 2). Fig. 5A shows the (normalised and smoothed) group activation in amHipp overlaid on the average structural MRI scan, accompanied by PHC and STS (Fig. 5B). To ensure that our object baselines engaged the expected regions, we also calculated the reverse contrast, which tested for greater activation for objects than scenes [(objects − scenes)]. This identified bilateral lateral occipital complex (LOC), a region commonly identified as responding to single objects, in addition to right lingual gyrus (Table 2).

##### 3.2.2.2 Imagination versus recall

We next examined how the response to scenes compared across tasks. We had hypothesised that imagining novel scenes and 1-week scene recall would engage similar brain regions, as both tasks involve constructing a scene representation from distributed elements across cortex. In support of this, a conjunction analysis (Table 3) found common activation for both tasks in regions of the core network: right amHipp, vmPFC, PHC, RSC and left STS [(imagine scenes − imagine objects) & (recall 1-week scenes − recall 1-week objects)]. Results for each condition individually are listed in Table 4. We then tested for differences between conditions. As expected, we found no significant results for imagining scenes versus 1-week recall [(imagine scenes − imagine objects) − (recall 1-week scenes − recall 1-week objects)] or the reverse contrast.

**Fig. 6:**
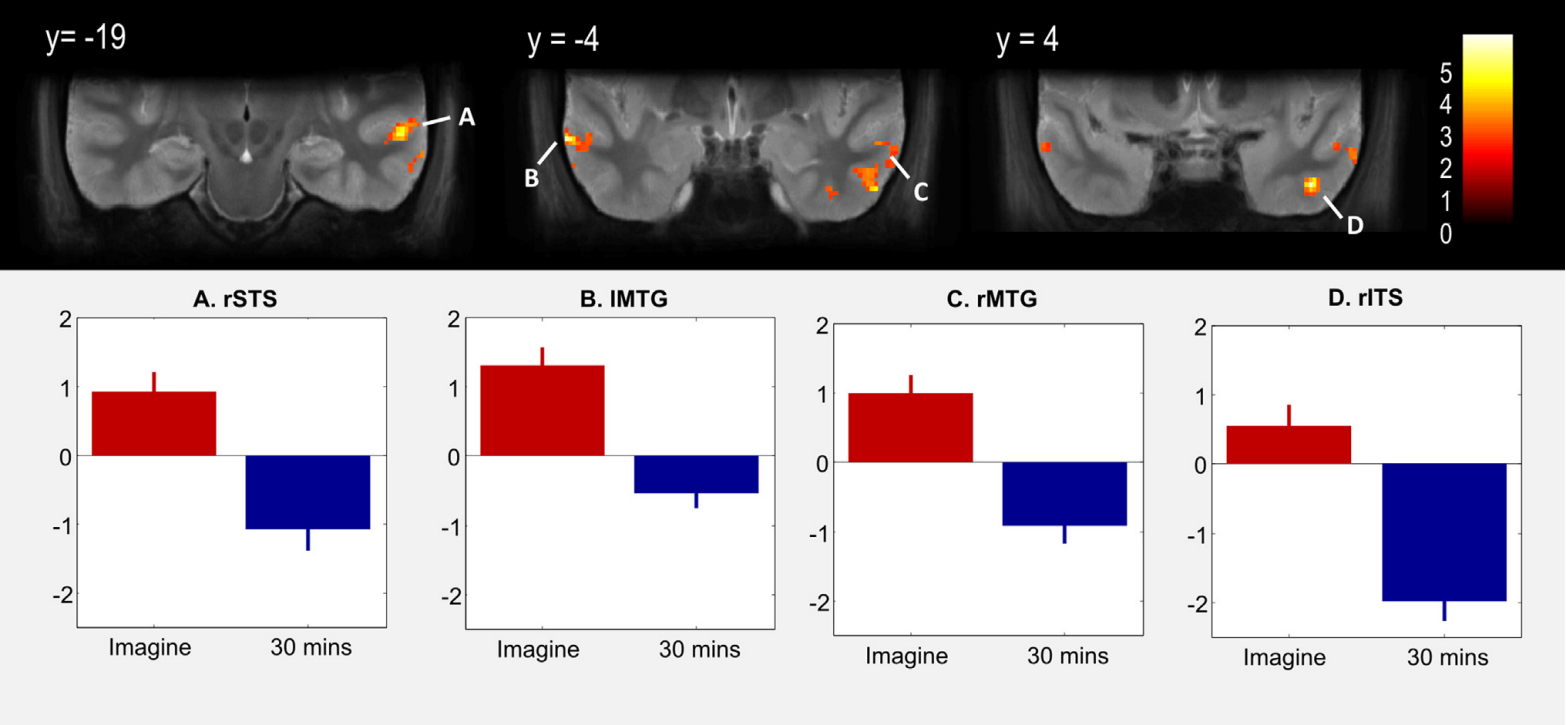
**Response to imagining novel scenes relative to 30-min scene recall**. SPM whole-volume analysis testing for regions responding more strongly to imagining novel scenes than recalling scenes from 30 min earlier, controlling for object baselines ([Imagine Scenes − Objects] − [30-Minute Scenes − Objects]), i.e., an interaction between stimulus and task. **Top**. Coronal slices showing clusters of activations in lateral temporal cortex relating to this interaction. Labels correspond to plots below. Images thresholded at *p* < .05 FDR-corrected, overlaid on the group average T2 structural volume. **Bottom**. Parameter estimates averaged over voxels in the corresponding clusters, describing the interaction in each region. Red bars show imagining novel scenes ([Imagine Scenes − Objects]) and blue bars show 30-min scene recall ([30-Minute Scenes − Objects]). Positive values indicate a stronger response to scenes than object baselines. ±1 SEM. Similar results pertained when 1-week scene recall was compared to 30-min scene recall (see Fig. S2). rSTS = right superior temporal sulcus, lMTG = left middle temporal gyrus, rMTG = right middle temporal gyrus, rITS = right inferior temporal sulcus.

We hypothesised that 30-min recall would cause less activation of regions involved with constructing scenes than the other tasks. We again performed a conjunction analysis, this time testing for regions engaged both by imagining novel scenes and recalling scenes from 30 min earlier, expecting fewer commonalities than found above [(imagine scenes − imagine objects) & (recall 30-min scenes − recall 30-min objects)]. Common activation was found only in bilateral RSC and PHC (Table 3), with no significant evidence for rest of the core network, such as vmPFC or lateral temporal cortices. To confirm whether any additional regions were engaged for 30-min recall, we examined the contrast for this condition alone [(recall 30-min scenes − recall 30-min objects)]. In addition to RSC and PHC, we found activation of a region of posterior hippocampus, likely reflecting pCA3 from the ROI analysis, and an anterior hippocampal activation likely reflecting PrS/PaS, as well as cerebellum (Table 4). To formally compare between imagining scenes and 30-min scene recall, we calculated the appropriate interaction between task and stimulus [(imagine scenes − imagine objects) − (recall 30-min scenes − recall 30-min objects)]. We found clusters in right STS, bilateral middle temporal gyrus (MTG) and right inferior temporal cortex (Fig. 6, Table 5). Plotting the parameter estimates demonstrated that each cluster had a greater response to scenes than objects in the imagine condition, whereas they had the opposite response in the 30-min recall condition (Fig. 6A–D). The interaction in right inferior temporal cortex was notable for being driven by a strong response to objects in the 30-min recall condition (Fig. 6D).

**Fig. S2:**
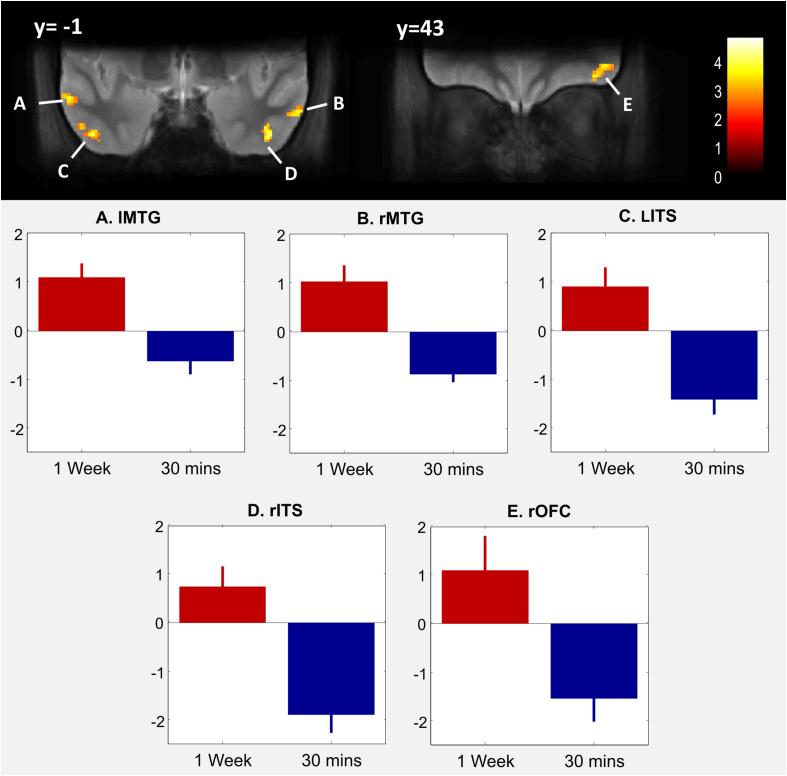
**Response to 1-week scene recall relative to 30-min scene recall.** SPM whole-volume analysis testing for regions responding more strongly to recalling scenes from one week earlier than scenes from 30 min earlier, controlling for object baselines [(1-week Scenes-Objects) − (30-Min Scenes-Objects)], i.e. an interaction between stimulus and task. **Top**. Coronal slices showing clusters of activations in lateral temporal cortex relating to this interaction. Labels correspond to plots below. Images thresholded at *p* < 0.05 FDR-corrected, overlaid on the group average T2 structural volume. **Bottom**. Parameter estimates averaged over voxels in the corresponding clusters, describing the interaction in each region. Red bars show 1-week scene recall [(1-Week Scenes-Objects)] and blue bars show 30-min scene recall [(30-Min Scenes-Objects)]. Positive values indicate a stronger response to scenes than object baselines. lMTG = left middle temporal gyrus, rMTG = right middle temporal gyrus, LITS = left inferior temporal sulcus, rITS = right inferior temporal sulcus, rOFC = right orbitofrontal cortex. ±1 SEM.

Having found reduced activity in lateral temporal cortex for 30-min scene recall compared to imagining novel scenes, we expected similar results when comparing 30-min scene recall to the 1-week recall condition [(1-week scenes − 1-week objects) − (30-min scenes − 30-min objects)]. This was the case (Table 5, Fig. S2), with significantly stronger activation for 1-week recall in lateral temporal cortex as well as right orbitofrontal cortex. The commonalities between 1-week and 30-min scene recall (Table 3) were only in PHC, RSC and right amHipp [(Recall 1-Week Scenes − Recall 1-Week Objects) AND (Recall 30-Minute Scenes − Recall 30-Minute Objects)]. For completeness, although we had not anticipated greater activation in the 30-min recall condition than the other conditions, we tested for this and found greater activation of cerebellum and primary visual cortex for 30-min recall than 1-week recall [(30-min scenes − 30-min objects) − (1-week scenes − 1-week objects)], potentially due to the marginally higher vividness ratings for 30-min recall. Only cerebellum had greater activation for 30-min recall than imagining novel scenes [(30-min scenes − 30-min objects) − (imagine scenes − imagine objects)].

In summary, we confirmed activation of the commonly observed ‘core’ network for scenes. We found this network to be engaged both by imagining novel scenes and recalling scenes from one week earlier. Recalling scenes from 30 min earlier also engaged three regions of this network, RSC and PHC and right amHipp, with significantly reduced activation in lateral temporal cortex.

## 4 Discussion

In this study we asked which structures within anterior hippocampus are engaged when internal representations of scenes are constructed. We found PrS/PaS responded when participants imagined novel scenes as well as when they recalled scenes which had been encoded 1 week or 30 min prior to scanning. Activation of other subregions of anterior hippocampus depended on whether the scenes were newly imagined or recalled from a week earlier. In contrast, recalling scenes from 30 min prior to scanning resulted in more restricted activation of the hippocampus, challenging the standard notion of systems consolidation. These findings help to link high level cognitive function to specific structures within hippocampal circuitry. By fractionating the wider ‘core’ network, we also help to explain what different regions may contribute to cognition.

### 4.1 Hippocampal subregions underlying scene processing

Many studies using scene or event stimuli, in the context of various experimental paradigms, have found a significant response in the medial portion of anterior hippocampus (e.g., Addis et al., 2007 [Fig. 1 their paper], Benoit & Schacter, 2015 [Fig 2, their paper], Hassabis, Kumaran, & Maguire, 2007 [Fig. 2, their paper], Zeidman et al., 2015 [Fig. 3 their paper]). Our first aim was to identify the precise hippocampal structures underlying this functional region. We found PrS/PaS to be the only structures engaged both by imagining novel scenes and recalling scenes from the past. What function might PrS/PaS serve?

In rats, dorsal (posterior) PrS and PaS contain a range of cell types that code for space. This includes place cells, which represent an animal's heading-invariant location (Taube, 1995), head-direction cells which represent place-invariant heading (Cacucci, Lever, Wills, Burgess, & O'Keefe, 2004), as well as conjunctive place-by-direction cells. More recently, PrS/PaS have been found to contain grid cells that fire at regular intervals over the environment (Boccara et al., 2010). This is in addition to conjunctive grid-by-heading and border cells, which fire when an animal is close to an environmental boundary. Far less attention has been given to ventral (anterior) PrS/PaS, which is more challenging to record in animals, however the extant findings suggest PrS/PaS could have the capacity to represent spatial properties of imagined scenes in humans.

In humans, several recent studies have capitalised on high resolution functional neuroimaging to investigate the hippocampus and connected structures. Maass et al. (2014) identified a peak of activity for visually perceiving novel scenes in the vicinity of PrS, which may have been representing the scenes while they were being perceived (Zeidman et al., 2015). Using functional connectivity analyses, Maass, Berron, Libby, Ranganath, and Düzel (2015) found distal subiculum, which borders on PrS, to have preferential connectivity with PHC – a region closely associated with scene processing (see also Libby, Ekstrom, Ragland, & Ranganath, 2012). They also found that posterior-medial EC, bordering on PaS, showed a similar profile. Although they did not separately segment PrS or PaS, their results suggest this region may have a preferential response to scenes due to its functional connectivity with PHC.

Understanding the role of PrS/PaS in humans may be helped by considering neuropsychological studies in patients with hippocampal lesions. Hassabis, Kumaran, Vann, et al. (2007) asked patients with bilateral hippocampal lesions and amnesia to describe fictitious atemporal scenes, such as “a sandy beach”. Evaluating patients' responses on a number of indices, they showed a specific deficit in spatial coherence; imagined scenes lacked richness because they were spatially fragmented. Similarly, Mullally et al. (2012) showed patients with hippocampal lesions a scene photograph and asked them to report what they would see if they stepped back from the depicted viewpoint in their imagination. While the patients' responses contained appropriate semantic content, their spatial detail was markedly reduced compared to controls, as was the vividness they reported of the imagined scene. These studies suggest that one contribution of the hippocampus to scenes may be spatial in nature. We propose that the tasks employed in this study – imagining novel scenes and recalling scenes from the past – engage the hippocampus in order to construct internal models or representations of scenes (Hassabis and Maguire, 2007, Maguire and Mullally, 2013). At the core of this proposal is a spatially coherent representation in the hippocampus which binds the scene's elements from across cortex, and our results suggest that anterior PrS/PaS may be key to the spatial aspect of this process.

Having investigated the overall response to scenes in the hippocampus, we next identified the similarities and differences between imagining novel scenes and recalling scenes from the past.

### 4.2 Comparing imagination and recall of scenes in the hippocampus

Our experiment included three experimental tasks – subjects imagined novel scenes, recalled scenes they had viewed one week before scanning and recalled scenes they had viewed 30 min before scanning. We hypothesised a similar response in the hippocampus for imagining novel scenes and recalling scenes from a week earlier, as we expected both to place similar demands on the process of scene construction. Although anterior hippocampus was engaged by both conditions as predicted, there were differences in the subregions engaged by each. Imagining novel scenes relative to single objects engaged aSub and PrS/PaS, whereas recalling scenes from a week earlier engaged the uncus and PrS/PaS. Unpacking these results, we first ask why was there a particular response in aSub to imagining scenes?

There were two distinct regions included in our mask for aSub – proximal or prosubiculum (ProS) and more distal subiculum proper, which lies further from DG. The hippocampal subfields project from DG to ProS then subiculum, which in turn projects to cortical and subcortical regions, and for this reason subiculum is considered the main output of the hippocampal subfields (O'Mara, 2006). It may operate to transform spatial codes in CA1 into compressed, information-rich codes suitable for transmission to other brain regions (Kim, Ganguli, & Frank, 2012). In a thorough review of subicular anatomy, Ding (2013) highlighted several features of the ProS and subiculum's connectivity which, we suggest, make them ideal candidates for involvement in constructing scenes. ProS has reciprocal connectivity with perirhinal cortex, which could convey objects and object novelty for inclusion in a scene (Murray & Richmond, 2001). Furthermore it projects to vmPFC; in this study we found vmPFC activation for constructing scenes as well as recalling scenes from a week earlier. It is possible that the enhanced response in vmPFC and other cortical regions was due to items retrieved from separate brain regions being assembled into a coherent scene representation in the hippocampus (Bonnici, Chadwick, Lutti, et al., 2012, Nieuwenhuis and Takashima, 2011). What vmPFC may contribute to memory and imagination is an open question, and there is not yet a consensus on this matter (Maguire, 2014). Also relevant to the connectivity of aSub are direct projections (in monkeys and rodents) from distal subiculum to the RSC and the mammillary bodies (Ding, 2013). These regions are involved in the head direction system, and together may represent and adjust the imagined heading in constructed scenes. Together, the available information on anatomical connectivity of subiculum suggests that transmission of spatial and object information may be of key importance to its function.

Next, we considered our finding that the 1-week recall condition engaged the uncus; indeed this was the only hippocampal subregion with greater activity for 1-week scene recall than imagining novel scenes. The uncus is a complex structure containing modified versions of the same subfields found in the main (intraventricular) hippocampus. Amaral, Insausti, and Cowan (1984) demonstrated that in monkeys, the DG of the uncus has direct commissural connections with the contralateral hippocampus, and these connections terminate specifically in the contralateral uncus. The uncus may therefore support inter-hemispheric connectivity. The subfields of the uncal hippocampus also have anatomical connections with other regions aligned with scene and object processing, including projections from uncal subiculum to the mammillary nucleus (Rosene & Van Hoesen, 1987) and uncal CA1 to perirhinal cortex and pre-frontal cortex (Insausti & Munoz, 2001). We did not have sufficient spatial resolution to distinguish individual subfields of the uncus, and further work is clearly needed to understand the functional implications of the modified subfields it contains.

The 30-min recall condition was associated with significantly less activation of aSub and uncus than the other conditions. One explanation is that this task was simply easier than the others, as reflected by participants' difficulty ratings. However, there is evidence that an interpretation based on difficulty is insufficient to explain our imaging results. Participants' difficulty ratings for the scene tasks were matched in the object baseline conditions, controlling for basic attentional effects. Furthermore, we found robust activation of PrS/PaS and pCA3 for the 30-min recall condition, demonstrating that 30-min recall did engage the hippocampus. In the wider brain there was also significant activation of RSC and PHC for 30-min recall, which we return to shortly. It is our proposal that the need to construct representations of scenes, either novel or recalled from a week earlier, taxed aSub and the uncus and contributed to the participants' sense of difficulty in those conditions. By contrast, in the 30-min recall condition, representations of the scenes had recently been constructed, leading to reduced activation of aSub and the uncus. All three conditions shared the requirement to represent the scenes such that they could be vividly experienced, which was supported by PrS/PaS.

Although this study focussed on explaining previous findings in anterior hippocampus, it was interesting that there was little or no evidence for activation in posterior hippocampus in the imagine condition (relative to the object baseline), whereas posterior regions pSub and pCA3 were engaged for 1-week and 30-min recall respectively. Why might recalling scenes have engaged posterior hippocampus? Poppenk et al. (2013) hypothesised that posterior hippocampus represents more fine-grained or detailed spatial information than the anterior. This may be relevant as stimuli in our recall conditions were encoded visually, unlike the imagine condition, and the vividness of scenes in the 30-min recall condition was rated as higher than the other conditions. That posterior hippocampus should respond to visual stimuli as well as recalled visual stimuli extends the recent finding that visual scene perception engages posterior hippocampus significantly more strongly than imagining scenes (Zeidman et al., 2015).

These results also speak to a wider debate on the role of the hippocampus in cognition. Standard consolidation theory (Squire & Alvarez, 1995) states that the hippocampus establishes a memory trace which is then fully transferred to the cortex for long-term storage, meaning the hippocampus has no involvement in the recall of remote memories. By this account, we might expect reduced hippocampal response for 1-week recall compared to 30-min recall, but that was not the case. We found PrS/PaS was engaged by both conditions, with greater activation of uncus for 1-week recall than 30-min recall. Our results complement the findings of Bonnici et al. (2013), who found that memories over longer time periods – 2 weeks and 10 years prior to scanning – could be decoded from the subfields of anterior hippocampus. Together, these results are better supported by models which propose involvement of the hippocampus in vivid recall in perpetuity, such as multiple trace theory (Nadel and Moscovitch, 1997, Winocur and Moscovitch, 2011) and the scene construction theory (Hassabis and Maguire, 2007, Maguire and Mullally, 2013).

### 4.3 The wider core network

Imagining and recalling scenes is known to engage a network of regions known as the ‘core’ network (Addis et al., 2009, Andrews-Hanna et al., 2010, Summerfield et al., 2010, Svoboda et al., 2006). We performed an analysis of the whole (partial) volume to identify the regions which co-activated with the hippocampal subfields identified above. For the main effect of scenes, PrS/PaS was accompanied by activation of bilateral PHC, RSC, vmPFC and lateral temporal cortex. A conjunction analysis showed this wide network to be engaged in common between imagining novel scenes and recalling scenes from a week earlier, supporting our hypothesis that a common process, which we suggest is scene construction, underpins both tasks. Although we found differences in uncus and aSub between imagination and 1-week scene recall, we did not find differences between these conditions in the whole-brain results.

The response to recalling scenes from 30 min prior to scanning had similarities and differences with the other conditions. A conjunction analysis of imagining novel scenes and recalling scenes from 30 min earlier (and similarly for the conjunction of 1-week and 30 min scene recall) found that all three scene tasks engaged PHC and RSC, regions of the core network associated with spatial scene processing. As all scene tasks included the requirement to vividly simulate scenes, these results suggest an ‘inner core’ network of PrS/PaS, PHC and RSC may support this process, which speaks to previous models of PHC and RSC function in spatial imagery (Byrne et al., 2007). The main differences we identified for 30-min recall were in lateral temporal cortices. Imagining novel scenes and recalling scenes from one week earlier each engaged regions of anterior STS and MTG, and there was a significantly reduced response in these regions during 30-min recall. What might STS and MTG contribute to the process of scene construction, which we suggest explains their increased involvement during imagination and 1-week recall of scenes?

This region has wide anatomical connectivity – within the core network it is directly connected with vmPFC and PHC, whereas more posterior STS is connected with RSC (Binder, Desai, Graves, & Conant, 2009). The anterior STS in particular has been implicated in autobiographical memory (Svoboda et al., 2006) and future thinking (Schacter, Addis, & Buckner, 2007). Anterior temporal cortex is particularly associated with semantic processing, and it may act as an amodal hub for linking together information of different modalities into unified concepts (Binder et al., 2009, Patterson et al., 2007, Ralph et al., 2010). Part of the definition of a scene is that it should be coherent (Maguire & Mullally, 2013), and as such when a scene is constructed, only relevant and meaningful items should be included. We may speculate that STS and MTG could provide the semantic information required to decide which elements make sense in the context of a scene. This process could be mediated by vmPFC, as the two regions have monosynaptic connectivity and lesions to vmPFC result in confabulation (Gilboa, Moscovitch, Baddeley, Kopelman, & Wilson, 2002), where patients spontaneously generate narratives of events that never occurred.

### 4.4 Summary and conclusions

We used fMRI to investigate the responses in hippocampal subregions and the wider brain to imagining and recalling scenes. Together, our results enable us to extend the ‘core’ network for scenes/memory to include specific subregions of the hippocampus, and propose a subdivision of this network into functional sub-networks. When imagining or recalling scenes, PrS/PaS is engaged together with PHC and RSC. These regions facilitate representation of the scenes and the production of simulated sensory imagery, as well as supporting the recall of scenes that have recently been encoded and have not yet been consolidated. However, if the scenes are recalled from consolidated memories or are newly constructed in the imagination, then vmPFC and lateral temporal cortex are also recruited, together with further subdivisions of the hippocampus (particularly aSub, pSub and uncus).

These results set clear directions for future work. First, what is it about scenes that cause certain regions within the hippocampus to be engaged more than single objects, and how exactly is this achieved? Second, it would help to know whether specific hippocampal subregions show a parametric increase in response with the age of memories being recalled. And third, what is the functional connectivity between the hippocampal subregions we have observed here and the rest of the brain? An analysis of this kind could help to better understand the role of the uncus, aSub and PrS/PaS in scene construction and scene recall.

## Figures and Tables

**Table 1:** Results of the post-scan memory tests.

	True Positives	True Negatives	False Positives	False Negatives
Constructed scenes	.88 (.10)	.98 (.04)	.01 (.04)	.11 (.10)
Constructed objects	.90 (.13)	.98 (.04)	.01 (.03)	.09 (.12)
Recalled 1-week scenes	.98 (.06)	1.00 (0)	.00 (0)	.02 (.06)
Recalled 1-week objects	.91 (.07)	.98 (.06)	.02 (.06)	.09 (.06)
Recalled 30-min scenes	.98 (.01)	.99 (.03)	.01 (.02)	.003 (.01)
Recalled 30-min objects	.99 (.02)	.96 (.05)	.03 (.05)	.003 (.01)

Mean rates are shown with standard deviations in brackets.

**Table 2:** Contrasts of scenes versus objects (whole volume).

Cluster peak/*sub-peak*	Extent (voxels)	Peak coordinates [*x y z*]	Peak *Z*
**Scenes > objects**			
Right PHC	1124	34 −37 −13	6.14
*Right amHipp*		23 −21 −18	4.08
Left RSC	1360	−10 −46 1	5.91
*Left PHC*		−21 −38 −14	5.79
*Left amHipp*		−21 −23 −18	5.03
*Left fusiform gyrus*		−25 −29 −25	3.65
Right RSC	245	9 −48 3	5.66
Left aSTS	284	−49 −5 −18	4.94
*Left aMTG*		−58 −13 −15	3.79
Right vmPFC	325	2 48 −12	4.19
Right aSTS	185	53 −5 −22	4.18
**Objects > scenes**			
Left LOC	1821	−46 −65 −5	5.04
*Left pITG*		−49 −49 −19	4.85
Right LOC	1308	41 −84 0	5.00
*Right pITG*		55 −57 −18	4.41
Right lingual gyrus	310	12 −60 −9	4.28

PHC = parahippocampal cortex, RSC = retrosplenial cortex, amHipp = anterior medial hippocampus, vmPFC = ventromedial prefrontal cortex, aSTS = anterior superior temporal sulcus, aMTG = anterior middle temporal gyrus, LOC = lateral occipital cortex, pITG = posterior inferior temporal gyrus.

**Table 3:** Conjunction analyses.

(Imagine scenes − imagine objects) AND (recall 1-week scenes − recall 1-week objects)			
Cluster peak/*sub-peak*	Extent (voxels)	Peak Coordinates [*x y z*]	Peak *Z*
Left RSC	218	−14 −51 5	6.25
Right PHC	843	33 −37 −13	5.99
*Right amHipp*		23 −21 −18	3.12
Left PHC	658	−21 −40 −13	5.90
Right RSC	309	12 −49 5	5.74
Left STS	159	−50 −4 −20	3.71
vmPFC	117	0 50 −10	3.66

(Imagine scenes − imagine objects) AND (recall 30-minute scenes − recall 30-minute objects)			
Cluster peak	Extent (voxels)	Peak Coordinates [*x y z*]	Peak *Z*
Right RSC	276	9 −48 3	6.00
Right PHC	709	33 −37 −13	5.99
Left PHC	596	−22 −40 −13	5.76
Left RSC	186	−14 −51 5	5.45

(Recall 1-week scenes − recall 1-week objects) AND (recall 30-minute scenes − recall 30-minute objects)			
Cluster peak/*sub-peak*	Extent (voxels)	Peak Coordinates [*x y z*]	Peak *Z*
Right PHC	880	33 −37 −13	6.57
*Right amHipp*		22 −21 −16	3.98
Left PHC	758	−27 −43 −9	6.53
Right RSC	274	11 −48 3	5.55
Left RSC	215	−14 −51 5	5.45

Abbreviations as for Table 2.

**Table 4:** Each scene condition versus its corresponding object condition.

Cluster peak/*sub-peak*	Extent (voxels)	Peak coordinates [*x y z*]	Peak *Z*
**Imagine scenes − imagine objects**			
Right PHC (lateral)	814	33 −37 −11	6.20
*Right PHC (medial)*		22 −37 −14	4.92
*Right amHipp*		22 −15 −25	4.78
Left PHC (lateral)	725	−28 −43 −7	5.66
*Left PHC (medial)*		−21 −40 −13	4.55
*Left amHipp*		−21 −20 −20	2.95
Left RSC	174	−14 −51 5	5.28
Right RSC	210	17 −46 1	5.20
Right vmPFC	318	5 47 −12	4.95
Left MTG	306	−61 2 −22	4.48
**Recall 1-week scenes − recall 1-week objects**			
Left PHC (medial)	1177	−19 −38 −16	5.96
*Left amHipp*		−19 −20 −20	3.94
Right PHC (medial)	1130	30 −38 −11	5.50
*Right amHipp*		23 −19 −15	4.13
Left RSC	203	−14 −51 5	5.30
Right RSC	205	9 −48 3	5.12
Left STS	255	−50 −5 −18	3.94
*Left MTG*		−60 −2 −20	3.39
**Recall 30-minute scenes − recall 30-minute objects**			
Left PHC (medial)	944	−28 −38 −13	5.65
*Left RSC*		−10 −49 1	4.83
*Left pHipp*		−28 −27 −11	2.87
Right PHC (lateral)	958	34 −35 −13	5.45
*Right amHipp*		20 −26 −16	4.98
*Right amygdala*		24 −5 −15	3.13
*Right pHipp*		34 −26 −11	3.13
*Right HATA*		19 −15 −13	3.09
*Right alHipp*		34 −18 −15	2.98
Right RSC	227	9 −46 5	5.04
Cerebellum	537	0 −68 −21	4.15

MTG = middle temporal gyrus, alHipp = anterior lateral hippocampus, HATA = hippocampal–amygdaloid transition area, other abbreviations as for Table 2.

**Table 5:** Interactions between conditions and stimuli (whole volume).

Interaction (imagine scenes − imagine objects) > (recall 30-minute scenes − recall 30-minute objects)			
Cluster peak/*sub-peak*	Extent (voxels)	Peak coordinates [*x y z*]	Peak *Z*
Right ITS	175	44 4 −37	4.30
*Right STS*		57 −4 −37	3.54
*Right ITG*		42 −1 −44	2.95
Left MTG	221	−65 −4 −18	4.07
Right STS	184	61 −19 −9	3.79
Right MTG	233	53 7 −25	3.42

Interaction (imagine scenes − imagine objects) > (recall 1-week scenes − recall 1-week objects)			
Cluster peak/*sub-peak*	Extent (voxels)	Peak coordinates [*x y z*]	Peak *Z*
	*None*		

Interaction (recall 1-week scenes − recall 1-week objects) > (imagine scenes − imagine objects)			
Cluster peak/*sub-peak*	Extent (voxels)	Peak coordinates [*x y z*]	Peak *Z*
	*None*		

Interaction (recall 30-minute scenes − recall 30-minute objects) > (imagine scenes − imagine objects)			
Cluster peak/*sub-peak*	Extent (voxels)	Peak coordinates [*x y z*]	Peak *Z*
Right cerebellum	923	17 −67 −24	4.81

Interaction (recall 30-minute scenes − recall 30-minute objects) > (recall 1-week scenes − recall 1-week objects)			
Cluster peak/*sub-peak*	Extent (voxels)	Peak coordinates [*x y z*]	Peak *Z*
Right cerebellum	420	1 −68 −21	4.29
Left calcarine sulcus	213	−3 −81 0	3.60

Interaction (recall 1-week scenes − recall 1-week objects) > (recall 30-minute scenes − recall 30-minute objects)			
Cluster peak/*sub-peak*	Extent (voxels)	Peak coordinates [*x y z*]	Peak *Z*
Left ITS	148	−47 −2 −37	3.75
*Left OTS*		−39 −10 −34	3.29
*Left ITG*		−54 1 −36	3.09
Left MTG	170	−58 −5 −17	3.66
Right OTS	198	41 −6 −37	3.66
*Right ITS*		46 1 −39	3.55
Right MTG	185	58 2 −29	3.55
Right OFC	176	35 36 −15	3.21

ITS = inferior temporal sulcus, ITG = inferior temporal gyrus, OTS = occipitotemporal sulcus STS = superior temporal sulcus, MTG = middle temporal gyrus, OFC = orbitofrontal cortex. Other abbreviations as for Table 2.
