# Supplementary material for: Investigating the functions of subregions within anterior hippocampus
Source: Cortex. 2015 Dec;73:240–56. doi: 10.1016/j.cortex.2015.09.002 (PMC4686003; doi:10.1016/j.cortex.2015.09.002)
Supplement: Supplementary file 1 [file mmc1.docx]

**Supplemental Material**

**Investigating the functions of subregions within anterior hippocampus**

Peter Zeidman, Antoine Lutti and Eleanor A. Maguire

**Table S1 – Planned post-hoc one sample t-tests on the main effect of Region**

| **Region** | **T** | **p** |
| --- | --- | --- |
| aDG | 1.247 | 0.229 |
| aCA3 | 0.785 | 0.443 |
| aCA1 | 0.099 | 0.923 |
| aSub | 1.881 | 0.077 |
| Uncus | 2.053 | 0.056 |
| **PrS/PaS** | **4.564** | **0.0004*** |
| pDG | 0.755 | 0.461 |
| pCA3 | 1.096 | 0.288 |
| pCA1 | 0.345 | 0.734 |
| pSub | 1.301 | 0.211 |

a=anterior, p=posterior, DG=dentate gyrus, Sub=subiculum, PrS/PaS=presubiculum/parasubiculum * p < 0.05 uncorrected, df=17

**Table S2 – Planned post-hoc paired samples t-tests on the Task x Stimulus interaction**

| **Condition** | **Region** | **T** | **p-value** |
| --- | --- | --- | --- |
| **Imagine vs 1 Week** | aDG | 1.486 | 0.155 |
|  | aCA3 | 1.886 | 0.077 |
|  | aCA1 | 0.449 | 0.659 |
|  | **aSub** | **2.276** | **0.036*** |
|  | **Uncus** | **2.134** | **0.048*** |
|  | PrS/PaS | 1.344 | 0.197 |
|  | pDG | 0.117 | 0.908 |
|  | pCA3 | 0.613 | 0.548 |
|  | pCA1 | 0.097 | 0.924 |
|  | pSub | 1.370 | 0.188 |
| **Imagine vs 30 Mins** | aDG | 1.366 | 0.190 |
|  | aCA3 | 0.573 | 0.574 |
|  | aCA1 | 1.245 | 0.230 |
|  | **aSub** | **2.648** | **0.017*** |
|  | Uncus | 0.644 | 0.528 |
|  | PrS/PaS | 1.372 | 0.188 |
|  | pDG | 1.133 | 0.273 |
|  | pCA3 | 0.642 | 0.529 |
|  | pCA1 | 0.209 | 0.837 |
|  | pSub | 0.046 | 0.964 |
| **1 Week vs 30 Mins** | **aDG** | **2.371** | **0.030*** |
|  | aCA3 | 1.242 | 0.231 |
|  | aCA1 | 1.528 | 0.145 |
|  | aSub | 1.389 | 0.183 |
|  | **Uncus** | **3.250** | **0.005*** |
|  | PrS/PaS | 0.369 | 0.717 |
|  | pDG | 1.565 | 0.136 |
|  | pCA3 | 2.081 | 0.053 |
|  | pCA1 | 0.074 | 0.942 |
|  | pSub | 1.867 | 0.079 |

Abbreviations as for Table S1. * p < 0.05 uncorrected, df=17
